# Are Published Cancer Care Trial Protocols With Traditional Chinese Medicine Interventions Concordant With SPIRIT-TCM Extension 2018? A Scoping Review on Published Trial Protocols Between 2019 and 2022

**DOI:** 10.1177/15347354231223966

**Published:** 2024-01-31

**Authors:** Hezheng Lai, Peiying Yang, Xin Shelley Wang, David Lim, Anderson Lam, Yucong Shi, Yishi Huang, Xiaoshu Zhu

**Affiliations:** 1 Chinese Medicine Centre (an international collaboration between Western Sydney University and Beijing University of Chinese Medicine), Western Sydney University, Campbelltown, NSW, Australia; 2 University of Texas, MD Anderson Cancer Centre, Houston, Houston, TX, USA; 3 University of Technology Sydney, Ultimo, NSW, Australia; 4 Mparntwe Center for Evidence in Health: A JBI Center of Excellence, Alice Spring, NT, Australia; 5 Jinan University, Guangzhou, Guangdong, China; 6 The University of Melbourne, Parkville, VIC, Australia; 7 School of Health Sciences, Western Sydney University, Campbelltown, NSW, Australia

**Keywords:** Traditional Chinese Medicine, acupuncture, Chinese Herbal Medicine, Tai Chi, Qigong, clinical trial, extension, recommendation, SPIRIT

## Abstract

**Background:**

The SPIRIT-TCM Extension 2018 was created to guide the design and reporting of Traditional Chinese Medicine (TCM) clinical trial protocols. This study aims to investigate the extent of concordance with this guideline in the relevant field of cancer care research.

**Methods:**

A scoping review of TCM cancer trial protocols published in English and Chinese since January 2019 was conducted. Five major academic databases (MEDLINE, EMBASE, CINAHL, CENTRAL, and China National Knowledge Infrastructure) were searched. Concordance with the SPIRIT-TCM Extension 2018 was assessed by descriptive analysis.

**Results:**

Fifty-three TCM cancer care trial protocols were identified, comprising 23 acupuncture, 26 Chinese herbal medicine (CHM), and 4 Tai Chi/Qigong (TCQ) interventions. The majority of the checklist items had a low rate of concordance, especially in the reporting of quality control and safety, dosage, TCM diagnostic patterns, possible interactions between Western Medicine and TCM interventions, and TCM-related outcome assessments.

**Conclusions:**

Although the SPIRIT-TCM Extension 2018 guideline was established through extensive Delphi consultation, there are low rates of concordance between published TCM cancer care clinical trial protocols with the guideline. Further research is necessary to understand the low rate of concordance and how scientific rigors of reporting can be improved in TCM cancer care research.

## Background

Cancer is one of the leading causes of death worldwide, thus optimizing the health and well-being of cancer survivors is a priority.^1^ Many cancer patients seek traditional Chinese medicine (TCM) to manage cancer and/or cancer treatment-related conditions to enhance cancer survival and to support health and well-being during and after cancer.^2^ TCM is one of the world’s oldest medical systems^3,4^ and has been increasingly adopted in Western countries.^5^ TCM is distinguished as a comprehensive whole-body system with a well-constructed standardized system of theory, containing a unique diagnostic framework and therapeutic practices.^6^ The most common TCM modalities represented in cancer care clinical practice and research include Chinese herbal medicine (CHM) formulas, acupuncture, and mind-body practices comprising Tai Chi and many forms of Qigong.^7^ Tai Chi and other forms of Qigong are grouped together for this review, consistent with other recent reviews, and collectively referred to as TCQ.^8-10^ Despite TCM having broad applications in China including cancer care,^11^ most developed countries have not officially recognized and adopted TCM. A paucity of reported scientific evidence is one of the perceived reasons.^7,12^ China conducts the greatest number of TCM clinical trials followed by the United States.^13^ Despite the increase in the amount of research being conducted in TCM interventions for cancer care,^14-18^ inconsistent study design, and reporting have undermined acceptance of results, therefore, impeding widespread acceptance of evidence-based TCM.^4^ Developing a comprehensive evidence-base for TCM application in cancer care is a priority for addressing this issue, whereas having a unified reporting standard is the one of the critical first steps. The SPIRIT-TCM Extension 2018 was developed for this purpose.^4,13-18^

The SPIRIT-TCM Extension 2018 was developed to address the challenges of applying SPIRIT 2013 Statement to trials involving TCM interventions, which have unique theories and characteristics. The traditional approach of TCM practice is usually individualized, which means the treatment, that is, acupuncture or CHM, will be varied according to the individual symptoms at a certain period. This means that the acupuncture protocol or CHM formulation will be changed. The “fixed” approach, more commonly expressed as the standardized approach, means that regardless of the time or individual, there is a singular approach that is applied to everyone unchanged. The SPIRIT-TCM Extension 2018 was developed as an extension to the original SPIRIT, to reflect the traditional approach of TCM practice and ensure the quality of trial design with TCM.

High-quality clinical trials are the foundation for establishing a system of clinical evidence. Every clinical trial should be based on high-quality protocols to ensure that the trial meets its objectives with rigor and efficiency. The Standard Protocol Items: Recommendations for Interventional Trials (SPIRIT) 2013 Statement was established and widely accepted by the international research community as an international standard for designing and reporting clinical trial protocols.^19^ Later in 2018, the Standard Protocol Items for Clinical Trials with Traditional Chinese Medicine 2018: Recommendations, Explanation, and Elaboration (SPIRIT-TCM Extension 2018) was developed through a comprehensive Delphi process. A total of 33 TCM researchers were surveyed, including TCM investigators, clinical research methodologists, statisticians, and clinical trial coordinators. The SPIRIT-TCM Extension 2018 is supposed to encompass the unique theory and characteristics of TCM interventions providing a more comprehensive guideline. It is currently not clear what the level of concordance with the SPIRIT TCM Extension 2018 guidelines is among protocols for cancer care trials involving TCM. Currently, a definitive standard of protocol design specific to TCM clinical trials in cancer care is also unknown. Moreover, there is no standard reporting guideline for TCQ interventions. With the increasing number of published study protocols, this scoping review intends to identify TCM cancer care clinical trial protocols published after the SPIRIT-TCM Extension 2018 guidelines and investigate their degree of concordance with these guidelines.

### Objectives

To report the extent of concordance with the SPIRIT-TCM Extension 2018 guidelines among the TCM cancer care clinical trial protocols published between 1st January 2019 to 23 September 2022 involving acupuncture, CHM, and TCQ interventions.

## Methods

A search was conducted on commonly used databases to extract information from clinical trial protocols for our review. Included protocols measured cancer outcomes in any disease stage and any cancer type and employed TCM interventions including (a) acupuncture, (b) CHM, or (c) TCQ interventions. The review was conducted in accordance with a prospectively registered protocol (DOI 10.17605/OSF.IO/ZCJYU).

### Search Strategy

Clinical trial protocols were identified by electronically searching the bibliographic databases MEDLINE (Ovid), EMBASE (EbscoHost), CINAHL (EbscoHost), Cochrane Central Register of Controlled Trials (CENTRAL) and the Chinese electronic database China National Knowledge Infrastructure (CNKI) using a search strategy formulated by an experienced medical librarian. All searches were performed independently by 2 investigators (HL and YS) for protocols published from 1st January 2019 to 23rd September 2022. A search strategy incorporating keywords and controlled vocabulary pertinent to our intervention (acupuncture OR Chinese herbal medicine OR qi gong OR tai chi [MeSH]) and outcome (cancer OR carcinoma OR breast neoplasms OR chemotherapy OR radiotherapy”[MeSH]) were used.

The retrieved published clinical trial protocols were imported into the reference management software EndNote™ 20.4.1 (Clarivate™). Two co-authors (HL and YS) independently screened all records by title and abstracts against the inclusion criteria. The same 2 authors then assessed the eligibility of relevant full-text protocols for inclusion in the review. Disagreements were resolved through discussion, with a third author (XZ) as arbiter where required. Consensus was reached on all inclusion. References of relevant protocols were also manually searched for eligible protocols that met the inclusion criteria. A final set of protocols fitting the scope of the present review underwent data extraction and data synthesis. Auditing of the data extraction was performed by 2 co-authors (HL and AL). The scoping review was conducted according to the Preferred Reporting Items for Systematic Reviews and Meta-Analyses Extension for Scoping Reviews (PRISMA-ScR) guidelines.^20^

### Eligibility Criteria

Protocols were included if they: (1) were clinical trial protocols designed for human subjects, (2) measured cancer outcomes in any disease stage and any cancer type; (3) employed TCM interventions including (a) acupuncture, (b) CHM, or (c) TCQ interventions; (4) were published in full text in a peer-reviewed journal from 1st January 2019 to 23rd September 2022; and (5) were published in English or Chinese language.

Protocols that focused on moxibustion and non-traditional acupuncture interventions (laser acupuncture, dry acupuncture, catgut embedding acupuncture, intradermal needle, or floating needle), Tuina (TCM massage), cupping, gua-sha, and intravenous injection were excluded.

The population for this review was cancer patients or survivors (regardless of gender, cancer, and treatment type) at any stage of their cancer trajectory (including advanced and palliative) receiving TCM interventions for cancer outcomes. TCM interventions included acupuncture, CHM, and TCQ. Acupuncture was defined as any type of traditional acupuncture intervention (manual acupuncture, electro-acupuncture, body acupuncture, auricular acupuncture). CHM interventions were limited to single herb and Chinese herbal formulas including fixed/standardized formulas and individualized formulas. The route of administration of CHM included oral and external use. The forms of administration included herbal decoctions, herbal extracts including powders/liquid/pills /tablets/capsules, and plasters. TCQ type, length of program, length of session, and frequency were not limited.

Cancer outcome measures included symptom management, quality of life (health-related quality of life, interference with daily living, and severity of symptoms and fatigue), survival (overall survival, progression-free survival, and disease/relapse-free survival), early efficacy endpoints (response rate, complete response, and duration of response), emerging efficacy endpoints (pathological complete response, immune-related response criteria, and minimal residual disease), safety (treatment side effects and treatment discontinuation rate), and end-of-life/survivorship.

### Data Extraction

Data from protocols were extracted to assess for concordance against all items in the SPIRIT-TCM Extension 2018 checklist^4^ and to review general protocol characteristics. General protocol characteristics included: (a) publication characteristics: title, year of publication, author, author’s affiliation country; (b) details of the intervention: acupuncture, CHM, and TCQ. A data extraction form was developed to collect the information necessary for data synthesis.

The data extraction form for assessment of concordance was developed based on the original 21 checklist items from the SPIRIT-TCM Extension 2018. Pilot testing of the data extraction form was performed by HL and AL on 5 included protocols. Each checklist item was assessed as either concordant, discordant, or unclear. Two authors (HL and AL) independently performed data extraction for all protocols. There was full agreement by the 2 authors on the independently extracted data, and inter-rater reliability was assessed by kappa statistic at 100%. Any discrepancies were planned to be discussed and solved by consensus by the 2 authors, with a third author (XZ) as arbiter where required.

### Data Analysis

Descriptive analysis of the concordance with SPIRIT-TCM Extension 2018 was performed by calculating the proportion (percentage) of checklist items that are concordant. Consistent with other studies, very low concordance is noted as ≤20%, low concordance as between 20% and 40%, fair concordance as between 40% and 60%, moderate concordance as between 60% and 80%, and good concordance at >80%.

## Results

**Figure 1.**
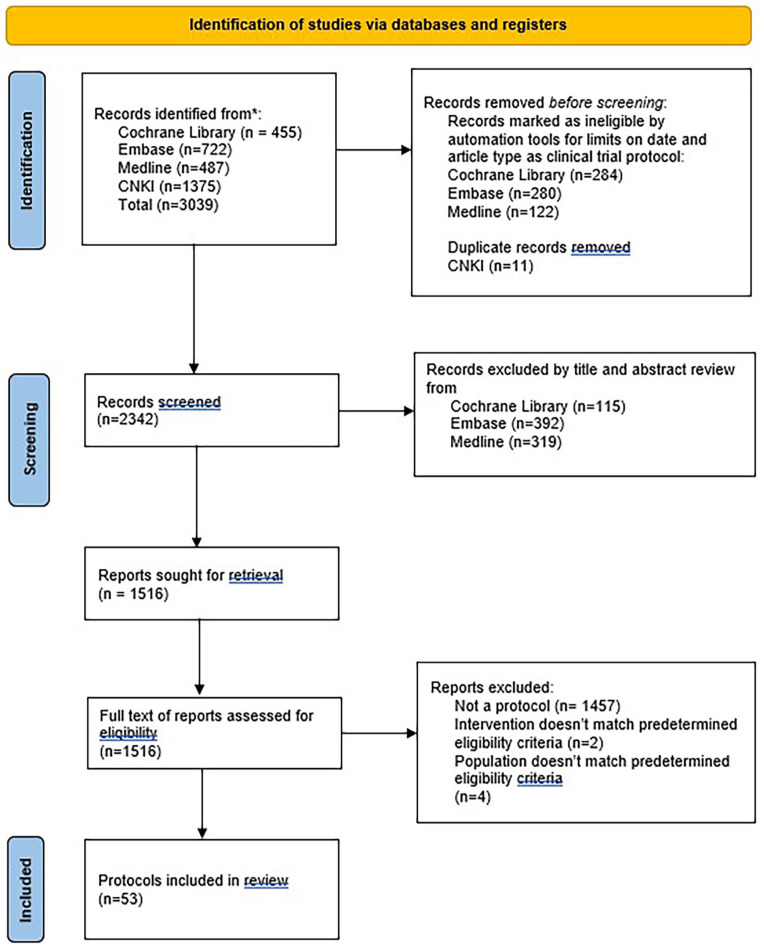
PRISMA flow diagram showing the search strategy and the number of included and excluded protocols.

A total of 53 protocols were retrieved. An initial search yielded 2342 articles from the 5 academic databases. After screening 2342 articles by title and abstract, 1516 articles remained. These articles were further screened as full text. A total of53 protocols were included in this review (see Figure 1).

### Included Protocol Characteristics

#### Region

Authors of the included protocols listed their affiliations in China, USA, Hong Kong, Australia, France, Germany, Korea, Spain, and Taiwan (Table 1). The majority of the protocols were from China (79%), followed by USA (9%). There were 8 protocols published in 2019, 15 protocols in 2020, 18 protocols in 2021, and 12 protocols from 1^st^ January 2022 up to 23^rd^ September 2022 (Table 3). Colorectal cancer is the predominant cancer type (25%), followed by breast (17%), lung (15%), gastric (11%), prostate (7%), cervical (2%), head and neck (2%), liver (2%), and oral cancer (2%); the rest did not specify a cancer type (Table 1). The majority of the cancer stages are Stage III (Table 1). The 53 protocols comprised 26 CHM (49%), 23 acupuncture (43%), and 4 TCQ intervention (8%) (Table 1). All included protocols were published in English. The majority of the protocols were published in the journal *Trials* (17/53, 32%) and *Medicin*e (Baltimore; 15/53, 28%). No protocols were published in Chinese peer-reviewed journals (Table 2).

**Table 1.** General Characteristics of Included Protocols.

Category	Number (%)
Year	China (42, 79); USA (5, 9); Hong Kong (3, 6); Australia (1, 2); France (1, 2); Germany (1, 2); Korea (1, 2); Spain (1, 2); and Taiwan (1, 2).
Cancer type	Colorectal (13, 25); open specification of cancer (12, 23); Breast (9, 17); Lung (8, 15); Gastric (6, 11); Prostate (3, 7); Cervical (1, 2); Head and Neck (1, 2); Liver (1, 2); and Oral Cancer (1, 2).
Cancer stage	Stage III (27, 51); open specification of staging (25, 47); Stage II (17, 32); Stage IV (12, 23); and Stage I (13, 25).
Intervention by grouping	CHM (26, 49); Acupuncture (23, 43); and TCQ (4, 8)
Interventions by specific therapy	CHM Decoction (13, 25); Body Acupuncture (12, 23); Electroacupuncture (9, 17); CHM Granule (7, 13); Auricular Acupuncture (3, 6); Tai Chi (3, 6); CHM Syrup (1, 2); External Use CHM (1, 2); CHM Capsule (1, 2); CHM Paste (1, 2); CHM Cream (1, 2); Baduanjin (1, 2); Hypoglossal Acupuncture (1, 2); and CHM with Food (1, 2).
Periodicals	Trials (17, 32); Medicine (Baltimore) (15, 28); Integrative Cancer Therapies (4, 8); BMJ (Open) (4, 8); Journal of Traditional Chinese Medicine, (2, 4); Annals of Palliative Medicine (1, 2); Chinese Journal of Integrative Medicine (1, 2); Contemporary Clinical Trials (1, 2); Contemporary Clinical Trials Communication (2, 4); Disease Markers (1, 2); European Journal of Integrative Medicine (1, 2); Evidence Based Complementary Alternative Medicine (1, 2); Frontiers Pharmacology (1, 2); Frontiers in Medicine (1, 2); and Translational Cancer Research (1, 2).

**Table 2.** Protocol Characteristics.

Author names	Year	Region	Intervention	Study center number	Sample size	Cancer
Wang et al^21^	2021	China	Acupuncture	6	168	Unrestricted
Chen et al^22^	2021	Hong Kong	Acupuncture	2	120	Unrestricted
Zhao et al^23^	2020	Australia	Acupuncture	3	106	Unrestricted
Yue et al^24^	2020	China	Acupuncture	3	252	Lung
Wang et al^25^	2020	China	Acupuncture	1	40	Gastrointestinal
Liou et al^26^	2020	USA	Acupuncture	1	360	Unrestricted
Lei et al^27^	2020	China	Acupuncture	1	120	Pancreatic
Lee et al^28^	2020	South Korea	Acupuncture	1	90	Colorectal/breast
Chan et al^29^	2020	Hong Kong	Acupuncture	1	84	Colorectal
Wang et al^30^	2019	China	Acupuncture	4	320	Lung
He et al^31^	2019	China	Acupuncture	1	15	Unrestricted
Haller et al^32^	2019	Germany	Acupuncture	1	25	Breast
Ben-Arie et al^33^	2019	China	Acupuncture	1	28	Oral
Zhang et al^34^	2021	China	CHM	1	120	Lung
Wang et al^20^	2021	China	CHM	1	160	Breast
Song et al^35^	2021	China	CHM	1	50	Colorectal or gastric
Pan et al^36^	2021	China	CHM	1	50	Liver
Yap et al^37^	2020	China	CHM	1	80	Unrestricted
Yao et al^38^	2020	China	CHM	5	354	Unrestricted
Xiao et al^39^	2020	China	CHM	1	50	Unrestricted
Wu et al^40^	2020	China	CHM	11	298	Gastric
Wei et al^41^	2020	China	CHM	13	360	Colorectal
Sun et al^42^	2020	China	CHM	6	450	Lung
Pan et al^43^	2020	China	CHM	6	210	Gastric
Deng et al^44^	2020	China	CHM	5	100	Lung
Zhou et al^45^	2019	China	CHM	4	130	Gastric
Zhou et al^46^	2019	China	CHM	7	400	Colorectal
Kinney et al^47^	2019	USA	TCQ	1	166	Unrestricted
Wei et al^48^	2021	China	TCQ	1	70	Breast
Shao et al^49^	2021	China	ACU	1	105	Colorectal
Zhang et al^50^	2021	Hong Kong	ACU	1	138	Breast
Liu et al^51^	2022	China	ACU	2	160	Colorectal
Lu et al^52^	2022	USA, South Korea, China	ACU	3	160	Breast
Lv et al^53^	2022	China	ACU	1	70	Breast
Mahasti et al^54^	2022	China, France, Spain	ACU	3	492	Breast
Romero et al^55^	2022	USA	ACU	7	284	Unrestricted
Wang et al^56^	2022	China	ACU	6	100	Unrestricted
Zhang et al^57^	2022	China	ACU	1	340	Prostate
Zou et al^58^	2022	China	ACU	4	248	Colorectal
Wang et al^59^	2020	China	CHM	1	66	Unrestricted
Chen et al^60^	2021	China	CHM	6	270	Lung
Gu et al^61^	2021	China	CHM	30	360	Colorectal
Hu et al^62^	2021	China	CHM	1	110	Cervical
Wang et al^63^	2021	China	CHM	3	150	Colorectal
Zhang et al^64^	2021	China	CHM	6	400	Colorectal
Zheng et al^65^	2021	China	CHM	1	168	Lung
Zhong et al^66^	2021	China	CHM	4	260	Colorectal
Cheng et al^67^	2022	Taiwan	CHM	1	40	Head and neck
Wu et al^68^	2022	China	CHM	1	80	Colorectal
Yao et al^38^	2022	China	CHM	7	314	Lung
Li et al^69^	2022	China	CHM	3	260	Gastrointestinal
Yao et al^70^	2021	China	MBT	2	72	Breast
Winters-Stone et al^71^	2021	USA	MBT	4	300	Prostate

## Overall Concordance

Table 3 shows the results for concordance with SPIRIT TCM 2018 Extension items in terms of the 3 interventions.

**Table 3.** Overall Concordance.

	Checklist item	Concordance			
		Total	ACU	CHM	TCQ
Category		N (%)	N (%)	N (%)	N (%)
Title	1a1. Specify the patient population in terms of 1) a WM-defined disease.	53/53 (100)	23/23 (100)	26/26 (100)	4/4 (100)
	1a2. Specify the patient population in terms of a WM-defined disease with a specific TCM Pattern.	2/53 (4)	0/23 (0)	2/26 (8)	0/4 (0)
	1a3. Specify the patient population in terms of a TCM Pattern.	2/53 (4)	0/23 (0)	2/26 (8)	0/4 (0)
	1b. Specify the intervention, in terms of (1) CHM formula, (2) acupuncture, (3) moxibustion, or (4) other TCM therapy(ies).	46/53 (87)	23/23 (100)	20/26 (77)	3/4 (75)
Background Rationale	6a.1 Provide the background and rationale of the research question with TCM theory.	8/53 (15)	2/23 (9)	6/26 (23)	0/4 (0)
	6a.2 Describe the rationale of the utilized TCM interventions with references.	19/53 (36)	12/23 (52)	5/26 (19)	2/4 (50)
	6a.3a Provide the rationale of adding experimental TCM interventions if WM intervention is used as basic or combined remedy.	21/53 (40)	9/23 (39)	9/26 (35)	3/4 (75)
	6a.3b Provide the potential interaction between WM intervention and TCM intervention (especially for CHM) explained with related reference(s).	2/53 (4)	2/23 (9)	0/26 (0)	0/4 (0)
	6b1. Describe the rationale and principle(s) for selecting comparators corresponding to certain interventions (ie, CHM formula, acupuncture, moxibustion, or other TCM interventions) considering comparable with tested intervention.	12/53 (23)	5/23 (22)	7/26 (27)	0/4 (0)
	6b2. Describe the rationale and principle(s) for selecting comparators corresponding to certain interventions (ie, CHM formula, acupuncture, moxibustion, or other TCM interventions) considering success of blinding.	3/53 (6)	1/23 (4)	2/26 (8)	0/4 (0)
Objectives	7a. State the objectives or hypotheses regarding the specific TCM intervention for a WM-defined disease.	53/53 (100)	23/23 (100)	26/26 (100)	4/4 (100)
	7b. State the objectives or hypotheses regarding the specific TCM intervention for a WM-defined disease with a specific TCM Pattern.	2/53 (4)	0/23 (0)	2/26 (8)	0/4 (0)
	7c. State the objectives or hypotheses regarding the specific TCM intervention for a TCM Pattern.	2/53 (4)	0/23 (0)	2/26 (8)	0/4 (0)
Eligibility criteria	10a1. State whether participants with a specific TCM Pattern will be recruited, in terms of 1) diagnostic criteria.	8/53 (15)	0/23 (0)	8/26 (31)	0/4 (0)
	10a2 Provide a universally recognized, or reference(s) with detailed explanation for the TCM Pattern diagnostic criteria.	7/53 (13)	0/23 (0)	7/26 (27)	0/4 (0)
	10a3 State whether participants with a specific TCM Pattern will be recruited, in terms of inclusion and exclusion criteria.	7/53 (13)	0/23 (0)	7/26 (27)	0/4 (0)
	10a4 Provide a universally recognized, or reference(s) with detailed explanation for the TCM Pattern inclusion and exclusion criteria.	6/53 (11)	0/23 (0)	6/26 (23)	0/4 (0)
	10b1. Descriptions of the roles, qualifications, and other relevant experience of the participant screeners.	3/53 (6)	1/23 (4)	2/26 (8)	0/4 (0)
	10b2. Descriptions of the roles, qualifications, and other relevant experience of the intervention providers.	19/53 (36)	18/23 (78)	0/26 (0)	1/4 (25)
	10b3. Descriptions of the roles, qualifications, and other relevant experience of the outcome assessors.	2/53 (4)	0/23 (0)	2/26 (8)	0/4 (0)
	10b4. Descriptions of the roles, qualifications, and other relevant experience of the data analysts.	0/53 (0)	0/23 (0)	0/26 (0)	0/4 (0)
	10c. Descriptions of the qualification and relevant experience of study center(s) involved in a TCM trial are recommended.	4/53 (8)	4/23 (17)	0/26 (0)	0/4 (0)
Interventions	Number of protocols with fixed CHM formula intervention			22	
	(fixed) 11a1A1. Name, source, and dosage form (eg, decoction, granules, powder, and pills).			5/22 (23)	
	(fixed) 11a1A2. Name, source, processing method, and dosage of each medical substance. Name of all substances should be presented in at least 2 types of languages: Chinese (Pinyin), Latin, or English. Names of the parts of the substances used should be specified.			8/22 (36)	
	(fixed) 11a1A3. Authentication method of each ingredient, and how, when, where, and by whom it will be conducted.			1/22 (5)	
	(fixed) 11a1A4. Production method of the formula.			0/22 (0)	
	(fixed) 11a1A5. Quality control of each ingredient and the whole formula.			2/22 (9)	
	(fixed) 11a1A6. Safety assessment of the formula, containing heavy metals and toxic elements test, pesticide residue test, microbial limit test, and acute/chronic toxicity test.			1/22 (5)	
	(fixed) 11a1A7. Dosage of the formula, and how the dosage was determined.			1/22 (5)	
	(fixed) 11a1A8. Administration route (eg, oral and external).			11/22 (50)	
	Number of protocols with individualized CHM formula intervention	3		3	
	(individualized ) 11a1A1. Name, source, and dosage form (eg, decoction, granules, powder, and pills).			0/3 (0)	
	(individualized ) 11a1A2. Name, source, processing method, and dosage of each medical substance. Name of all substances should be presented in at least 2 types of languages: Chinese (Pinyin), Latin, or English. Names of the parts of the substances used should be specified.			0/3 (0)	
	(individualized ) 11a1A3. Authentication method of each ingredient, and how, when, where, and by whom it will be conducted.			0/3 (0)	
	(individualized ) 11a1A4. Production method of the formula.			0/3 (0)	
	(individualized ) 11a1A5. Quality control of each ingredient and the whole formula.			0/3 (0)	
	(individualized ) 11a1A6. Safety assessment of the formula, containing heavy metals and toxic elements test, pesticide residue test, microbial limit test, acute/chronic toxicity test.			0/3 (0)	
	(individualized ) 11a1A7. Dosage of the formula, and how the dosage was determined.			0/3 (0)	
	(individualized ) 11a1A8. Administration route (eg, oral, external).			1/3 (33)	
	(individualized ) 11a1A9. Additional information: how, when, and by whom the formula will be modified.			1/3 (33)	
	Number of protocols with patent CHM Formula intervention			2	
	(patent) 11a.1A1 Details of reference to a publicly available material(s), such as Pharmacopeia, for the details about the composition of the formula.			1/2 (50)	
	(patent) 11a.1A2 Details of reference to a publicly available material(s), such as Pharmacopeia, for the details about the dosage of the formula.			1/2 (50)	
	(patent) 11a.1A3 Details of reference to a publicly available material(s), such as Pharmacopeia, for the details about the efficacy of the formula.			0/2 (0)	
	(patent) 11a.1A4 Details of reference to a publicly available material(s), such as Pharmacopeia, for the details about the safety of the formula.			0/2 (0)	
	(patent) 11a.1A5 Details of reference to a publicly available material(s), such as Pharmacopeia, for the details about the quality control of the formula.			1/2 (50)	
	(patent) 11a.1A6 Detail of the proprietary product name (ie, brand name)			2/2 (100)	
	(patent) 11a.1A7 Detail of name of manufacturer.			2/2 (100)	
	(patent) 11a.1A8 Detail of lot number.			1/2 (50)	
	(patent) 11a.1A9 Detail of production data and expiry date.			0/2 (0)	
	(patent) 11a.1A10 Detail of name and content of added materials			1/2 (50)	
	(patent) 11a.1A11 Detail of whether any additional quality control procedures will be conducted.			0/2 (0)	
	(patent) 11a.1A12 Statement of whether the patent proprietary CHM formula utilized in the study is identical to the publicly available reference.			0/2 (0)	
	Number of protocols with acupuncture intervention		23		
	11a.1B1. Detail of treatment environment.		11/23 (48)		
	11a.1B1A. Detail of participant posture.		11/23 (48)		
	11a.1B2. Detail of number of needle insertions per subject per session (mean and range if possible).		17/23 (74)		
	11a.1B3. Detail of location of acupoints (uni/bilateral).		13/23 (57)		
	11a.1B3A. Name of all acupoints presented in Chinese (Pinyin) and international code.		16/23 (70)		
	11a.1B4. Detail of angle of insertion.		3/23 (13)		
	11a.1B4A. Detail of depth of insertion, which should be presented in a specified unit of measurement or on a particular tissue level.		12/23 (52)		
	11a.1B5. Detail of response sought (eg, de qi or muscle twitch response).		19/23 (83)		
	11a.1B6. Detail of needle stimulation (eg, manual and electrical). If electroacupuncture apparatus will be utilized, the brand, manufacturer, and frequency should be indicated.		16/23 (70)		
	11a.1B7. Detail of needle retention time.		21/23 (91)		
	11a.1B8A. Detail of needle, including diameter and length.		21/23 (91)		
	11a.1B8B. Detail of manufacturer.		16/23 (70)		
	11a.1B8C. Detail of needle material.		10/23 (43)		
	11a.1B9. Detail of number of treatment sessions.		23/23 (100)		
	11a.1B10. Detail of frequency and duration of treatment sessions.		22/23 (96)		
	Number of protocols that used placebo control			22	
	Placebo control 11a.2A1 Name and dosage of each ingredient.			4/22 (18)	
	11a.2A2. Description of the similarity of placebo with intervention (eg, color, smell, taste, appearance, packing).			17/22 (77)	
	11a.2A3. Quality control and safety surveillance, if any.			2/22 (9)	
	11a.2A4. Administration route, dosage, and regimen.			11/22 (50)	
	11a.2A5. Production information: when, where, how, and by whom the placebo was produced.			1/22 (5)	
	Number of protocols with CHM active control	0	0	0	0
	CHM active control 11a.2A1 If a CHM formula was used, refer to the recommendations of 11a.1A.				
	Pharmacological active control Number of protocols that used a chemical agent as control		0	10	0
	Pharmacological active control 11a.2A2 If a chemical agent was used, the name.			10/10 (100)	
	11a.2A3 If a chemical agent was used, the administration route.			3/10 (30)	
	11a.2A4 If a chemical agent was used the dosage			8/10 (80)	
	11a.2A5 If a chemical agent was used the regime.			10/10 (100)	
	11a.2B1. Acupuncture Blank/waitlist control State any special arrangement(s) in pre-treatment, treatment, and post-treatment periods corresponding to the experimental intervention (eg, examinations in pre-treatment period, unaltered lifestyle and medication in treatment period, and compensatory interventions in post-treatment period).		7/10 (70)		
	11a.2B2. Acupuncture Sham acupuncture or acupuncture-like control State the comparability of the sham acupuncture or acupuncture-like control and comprehensively provide details as for the recommendations of 11a.1B.		7/12 (58)		
	11d.2 Descriptions of other interventions that will be administrated to experimental and/or control groups are recommended (eg, rescue interventions), with enough details to allow replication.	16/53 (30)	12/23 (52)	4/26 (15)	0/4 (0)
Outcomes	12a. Provide the rationale of TCM-related indexes as outcomes (eg, the change of degree and scope of symptoms and signs related to pattern differentiation).	7/53 (13)	0/23 (0)	7/26 (27)	0/4 (0)
	12b1. Provide the details of the TCM-related outcomes assessment, including the measuring methods and standard (eg, frequency, severity rating scale of symptoms and signs, verified Pattern questionnaire, time points for assessment, and corresponding rationale.)	7/53 (13)	0/23 (0)	7/26 (27)	0/4 (0)
	12b2. Provide the details of the TCM-related outcomes assessment including assessor qualification (eg, relevant assessment experience, years in clinical practice).	3/53 (6)	0/23 (0)	3/26 (12)	0/4 (0)
	12b3. Provide the details of the TCM-related outcomes assessment including methods used to enhance the quality of assessment (eg, multiple repeated observation, training of assessors).	5/53 (9)	0/23 (0)	5/26 (19)	0/4 (0)
	18a.1 Protocol was targeted on TCM Pattern, or a WM-defined disease with a specific TCM Pattern baseline data about TCM Pattern was provided.	6/53 (11)	0/23 (0)	6/26 (23)	0/4 (0)
Data collection methods	31a1. Plan for raw data sharing with detail of when the data will become available.	3/53 (6)	2/23 (9)	1/26 (4)	0/4 (0)
	31a2. Plan for raw data sharing with detail of how the data will be shared.	4/53 (8)	3/23 (13)	1/26 (4)	0/4 (0)
Dissemination Policy	31a3. Plan for raw data sharing with detail of what data in particular will be shared.	5/53 (9)	4/23 (17)	1/26 (4)	0/4 (0)
	31a4. Plan for raw data sharing with detail of who could acquire the data.	9/53 (17)	7/23 (30)	2/26 (8)	0/4 (0)
	31a5. Plan for raw data sharing with detail of through what access data will be shared.	2/53 (4)	2/23 (9)	0/26 (0)	0/4 (0)

### Title

There was full concordance with specifying the patient population in terms of a Western Medicine (WM) defined disease (cancer). However, there was very low concordance at 4% (2/53) with specifying the patient population in terms of a TCM diagnostic pattern. Only 2 protocols, both CHM intervention protocols, included the TCM diagnostic pattern.^46,63^ There was good concordance at 87% (46/53) with specifying the intervention in terms of a specific TCM modality. There were 6 CHM and 1 TCQ intervention protocol that did not specify the TCM modality in the title (Table 3).

### Background and Rationale

There was very low concordance at 15% (8/53) with providing TCM theory for the rationale of the research question, with the lowest concordance found in TCQ protocols at 0%, followed by acupuncture protocols at 9% (2/23). There was low concordance at 36% (19/53) in reporting the rationale for utilizing TCM interventions, and at 40% (21/53) in reporting the rationale for combining TCM and WM intervention as a basic or combined remedy. There was very low concordance at 4% (2/53) with reporting the potential interaction between WM and TCM intervention, with the lowest concordance found in both TCQ and CHM protocols at 0%. There was low concordance at 23% (12/53) with specifying the rationale for selecting comparators that correspond to TCM modalities, with the lowest concordance found in TCQ protocols at 0%. There was very low concordance at 6% (3/53) in specifying the rationale for comparators in terms of the success of blinding, with the lowest concordance found in TCQ protocols at 0%.

### Objectives

There was good concordance, at 100% (53/53) in all protocols, with specifying the objectives regarding the intervention for a WM-defined disease. However, there was very low concordance at 4% (2/53) with specifying the objectives regarding the intervention for a TCM diagnostic pattern, with the lowest concordance found in both acupuncture and TCQ protocols at 0%.

### Eligibility Criteria

There was very low concordance at 15% (8/53) with specifying and referencing diagnostic criteria for TCM diagnostic pattern, with the lowest concordance found in both acupuncture and TCQ protocols at 0%. There was very low concordance at 13% (7/53) with specifying TCM diagnostic patterns as part of the participant inclusion and exclusion criteria, with the lowest concordance found in both acupuncture and TCQ protocols at 0%.

There was very low concordance with describing the qualification, and experience of participant screeners at 6% (3/53), outcome assessors at 4% (2/53), data analysts at 0% (0/53), and the study center(s) at 8% (4/53), and low concordance with reporting TCM intervention providers at 36% (19/53). TCQ protocols had by far the lowest rate of concordance across all 4 aforementioned items at 0% respectively.

### Interventions

#### Standardized CHM intervention

There were 22 protocols with fixed CHM intervention. The overall concordance rate was very low, notably in reporting the formula’s profile in safety and quality. There was very low concordance at 5% (1/22) with reporting the formula’s safety assessment,^37^ 9% (2/22) with reporting the quality control of each ingredient and the formula as a whole,^20,37^ 0% (0/22) with reporting the production method of the formula, and 5% (1/22) with reporting the authentication method of each ingredient.^37^ There was very low concordance with reporting the formula’s details of administration such as dosage at 5% (1/22), fair concordance with reporting administration route at 50% (11/22), low concordance with reporting dosage form at 23% (5/22), and low concordance with reporting substance name 36% (8/22).

#### Individualized CHM intervention

There were 3 protocols with individualized CHM intervention. The overall concordance rate was very low, particularly in reporting the formula’s profile in safety, quality, and administration details. There was 0% concordance with reporting the formula’s safety assessment, quality control, production method, and the authentication method of each ingredient. There was 0% concordance with reporting the formula’s details of administration, dosage, dosage form, administration route, and substance name. There was low concordance with reporting formula modification details at 33% (1/3).

#### Patent CHM intervention

There were 2 protocols with patent CHM intervention. The overall concordance rate ranged from low to good across the different checklist items. There was good concordance at 100% (2/2) with reporting the name of the manufacturer and the proprietary product name. There was fair concordance at 50% (1/2) with reporting formula composition, dosage, and reference to publicly available materials (such as Pharmacopeia) for details about quality control. There was 0% (0/2) concordance with making reference to publicly available materials (such as Pharmacopeia) about the safety and efficacy of the formula, and in reporting production data, formula expiry date, and additional quality control procedures.

#### Acupuncture intervention

There were 23 protocols with acupuncture intervention and the overall concordance rate ranged from moderate to very good with reporting intervention administration. In administration details, there was good concordance at 100% (23/23) with reporting the detail of treatment sessions, 96% with reporting the frequency and duration of treatment sessions (22/23), 91% (21/23) with reporting the needle retention time, 83% (19/23) with reporting the detail of acupuncture response sought, and moderate concordance with reporting the detail of either manual or electrical stimulation at 70% (16/23). There was very good concordance at 91% (21/23) with reporting of details of the needle including diameter and length, and moderate concordance at 70% (16/23) with reporting the detail of the needle manufacturer. However, there was very low concordance with reporting the angle of needle insertion (13%), and fair concordance with reporting the detail of needle material (43%), depth of needle insertion (52%), detail of treatment environment (48%) and participant posture (48%).

#### CHM placebo control

There were 22 protocols with CHM placebo control. There was moderate concordance with the reporting of the similarity of placebo with intervention at 77% (17/22) and fair concordance with reporting the administration route, dosage, and regimen at 50% (11/22). However, there was very low concordance with reporting the name and dosage of each placebo ingredient at 18% (4/22), the quality and safety surveillance at 9% (2/22), and production information of the placebo at 5% (1/22).

#### CHM active control

None of the CHM protocols used a CHM formula as an active control.

#### Pharmacological active control

There were 10 protocols that used pharmacological active control with mostly good concordance across the items. There was good concordance at 100% with reporting the name and regime of the chemical agent, and at 80% with reporting the dosage of the chemical agent. However, there was low concordance at 30% with reporting the administration route of the chemical agent.

#### Blank/waitlist control

There were 10 out of 23 protocols that used blank/waitlist control. There was moderate concordance with reporting the arrangement of the control in correspondence to the intervention at 70% (7/10).

#### Sham acupuncture or acupuncture-like control

There were 12 out of 23 protocols that used sham acupuncture or acupuncture-like control. There was fair concordance with reporting the comparability of the control with the intervention at 58% (7/12).

#### Descriptions of other interventions administrated

There was low concordance at 30% (16/53) with reporting details of other interventions used alongside intervention and/or control groups with enough details to allow replication, with CHM protocols at 15% and the lowest concordance found in TCQ protocols at 0% .

### Outcomes

There was very low concordance with reporting TCM-related outcome assessments (15%, 7/53), the corresponding assessor qualifications (6%, 3/53) and methods to enhance assessment quality (9%, 5/53). The lowest concordance was found in acupuncture and TCQ protocols at 0% across the above items.

### Data Collection Methods

There was very low concordance at 11% (6/53) with providing baseline data about TCM diagnostic patterns, and again the lowest concordance was found in acupuncture and TCQ protocols at 0%.

### Dissemination Policy

There was very low concordance with reporting the detail of data availability at 6% (3/53), method of data sharing at 8% (4/53), particular data shared at 9% (5/53), who may acquire the data at 17% (9/53), and through what access data will be shared at 4% (2/53). The lowest concordance was found in TCQ protocols at 0% (0/4) across all the checklist items.

### Sensitivity Analysis: Assessment of Plausible Time Delay Effect

To investigate whether the above poor concordance rate was due to the time delay effect in terms of the publication of the SPIRIT-TCM Extension 2018, we also reviewed the rates of concordance by year of publication. We found that rates of concordance remained quite low across the years 2019 to 2022, and we did not observe a remarkable difference in improvement in the protocols published in the subsequent years. Table 4 shows the results for concordance with SPIRIT TCM 2018 Extension by year of protocol publication.

**Table 4.** Concordance by Year of Protocol Publication.

	Checklist item	Concordance			
		2019	2020	2021	2022
Category		N (%)	N (%)	N (%)	N (%)
Title	1a1. Specify the patient population in terms of 1) a WM-defined disease.	7/8 (14)	15/15 (100)	17/18 (94)	12/12 (100)
	1a2. Specify the patient population in terms of a WM-defined disease with a specific TCM Pattern.	1/8 (13)	0/15 (0)	1/18 (6)	0/12 (0)
	1a3. Specify the patient population in terms of a TCM Pattern.	0/8 (0)	0/15 (0)	0/18 (0)	0/12 (0)
	1b. Specify the intervention, in terms of (1) CHM formula, (2) acupuncture, (3) moxibustion, or (4) other TCM therapy(ies).	7/8 (88)	13/15 (87)	15/18 (83)	11/12 (92)
Background rationale	6a.1 Provide the background and rationale of the research question with TCM theory.	1/8 (13)	3/15 (20)	2/18 (11)	2/12 (17)
	6a.2 Describe the rationale of the utilized TCM interventions with references.	4/8 (50)	7/15 (47)	4/18 (22	4/12 (33)
	6a.3a Provide the rationale of adding experimental TCM interventions if WM intervention is used as basic or combined remedy.	2/8 (25)	3/15 (20)	7/18 (39)	9/12 (75)
	6a.3b Provide the potential interaction between WM intervention and TCM intervention (especially for CHM) explained with related reference(s).	0/8 (0)	1/15 (7)	0/18 (0)	1/12 (8)
	6b1. Describe the rationale and principle(s) for selecting comparators corresponding to certain interventions (ie, CHM formula, acupuncture, moxibustion, or other TCM interventions) considering comparable with tested intervention.	3/8 (38)	2/15 (13)	3/18 (17)	4/12 (33)
	6b2. Describe the rationale and principle(s) for selecting comparators corresponding to certain interventions (ie, CHM formula, acupuncture, moxibustion, or other TCM interventions) considering success of blinding.	1/8 (13)	1/15 (7)	1/18 (6)	0/12 (0)
Objectives	7a. State the objectives or hypotheses regarding the specific TCM intervention for a WM-defined disease.	7/8 (88)	15/15 (100)	17/18 (94)	12/12 (100)
	7b. State the objectives or hypotheses regarding the specific TCM intervention for a WM-defined disease with a specific TCM Pattern.	1/8 (13)	0/15 (0)	1/18 (6)	0/12 (0)
	7c. State the objectives or hypotheses regarding the specific TCM intervention for a TCM Pattern.	0/8 (0)	0/15 (0)	0/18 (0)	0/12 (0)
Eligibility criteria	10a1. State whether participants with a specific TCM Pattern will be recruited, in terms of 1) diagnostic criteria.	1/8 (13)	4/15 (27)	2/18 (11)	1/12 (8)
	10a2 Provide a universally recognized, or reference(s) with detailed explanation for the TCM Pattern diagnostic criteria.	1/8 (13)	3/15 (20)	2/18 (11)	1/12 (8)
	10a3 State whether participants with a specific TCM Pattern will be recruited, in terms of inclusion and exclusion criteria.	1/8 (13)	3/15 (20)	2/18 (11)	1/12 (8)
	10a4 Provide a universally recognized, or reference(s) with detailed explanation for the TCM Pattern inclusion and exclusion criteria.	1/8 (13)	2/15 (13)	2/18 (11)	1/12 (8)
	10b1. Descriptions of the roles, qualifications and other relevant experience of the participant screeners.	0/8 (0)	3/15 (20)	0/18 (0)	0/12 (0)
	10b2. Descriptions of the roles, qualifications and other relevant experience of the intervention providers.	5/8 (63)	5/15 (33)	2/18 (11)	7/12 (58)
	10b3. Descriptions of the roles, qualifications, and other relevant experience of the outcome assessors.	0/8 (0)	1/15 (7)	1/18 (6)	0/12 (0)
	10b4. Descriptions of the roles, qualifications, and other relevant experience of the data analysts.	0/8 (0)	0/15 (0)	0/18 (0)	0/12 (0)
	10c. Descriptions of the qualification and relevant experience of study center(s) involved in a TCM trial are recommended.	1/8 (13)	2/15 (13)	0/18 (0)	1/12 (8)
Interventions	Number of protocols with fixed CHM formula intervention	3	6	10	3
	(fixed) 11a1A1. Name, source, and dosage form (eg, decoction, granules, powder, and pills).	1/3 (33)	1/6 (17)	2/10 (20)	1/3 (33)
	(fixed) 11a1A2. Name, source, processing method and dosage of each medical substance. Name of all substances should be presented in at least 2 types of languages: Chinese (Pinyin), Latin, or English. Names of the parts of the substances used should be specified.	1/3 (33)	4/6 (67)	2/10 20	1/3 (33)
	(fixed) 11a1A3. Authentication method of each ingredient, and how, when, where, and by whom it will be conducted.	0/3 (0)	1/6 (17)	0/10 (0)	0/3 (0)
	(fixed) 11a1A4. Production method of the formula.	0/3 (0)	0/6 (0)	0/10 (0)	0/3 (0)
	(fixed) 11a1A5. Quality control of each ingredient and the whole formula.	0/3 (0)	1/6 (17)	1/10 (10)	0/3 (0)
	(fixed) 11a1A6. Safety assessment of the formula, containing heavy metals and toxic elements test, pesticide residue test, microbial limit test, acute/chronic toxicity test.	0/3 (0)	1/6 (17)	0/10 (0)	0/3 (0)
	(fixed) 11a1A7. Dosage of the formula, and how the dosage was determined.	0/3 (0)	1/6 (17)	0/10 (0)	0/3 (0)
	(fixed) 11a1A8. Administration route (eg, oral, external).	1/3 (33)	2/6 (33)	5/10 (50)	3/3 (100)
	Number of protocols with individualized CHM formula intervention		2		1
	(individualized ) 11a1A1. Name, source, and dosage form (eg, decoction, granules, powder, and pills).		0/2 (0)		0/1 (0)
	(individualized ) 11a1A2. Name, source, processing method and dosage of each medical substance. Name of all substances should be presented in at least 2 types of languages: Chinese (Pinyin), Latin, or English. Names of the parts of the substances used should be specified.		0/2 (0)		0/1 (0)
	(individualized ) 11a1A3. Authentication method of each ingredient, and how, when, where, and by whom it will be conducted.		0/2 (0)		0/1 (0)
	(individualized ) 11a1A4. Production method of the formula.		0/2 (0)		0/1 (0)
	(individualized ) 11a1A5. Quality control of each ingredient and the whole formula.		0/2 (0)		0/1 (0)
	(individualized ) Safety assessment of the formula, containing heavy metals and toxic elements test, pesticide residue test, microbial limit test, acute/chronic toxicity test.		0/2 (0)		0/1 (0)
	(individualized ) 11a1A7. Dosage of the formula, and how the dosage was determined.		0/2 (0)		0/1 (0)
	(individualized ) 11a1A8. Administration route (eg, oral, external).		0/2 (0)		1/1 (100)
	(individualized ) 11a1A9. Additional information: how, when, and by whom the formula will be modified.		1/2 (50)		0/1 (0)
	Number of protocols with patent CHM Formula intervention			2	
	(patent) 11a.1A1 Details of reference to a publicly available material(s), such as Pharmacopeia, for the details about the composition of the formula.			1/2 (50)	
	(patent) 11a.1A2 Details of reference to a publicly available material(s), such as Pharmacopeia, for the details about the dosage of the formula.			1/2 (50)	
	(patent) 11a.1A3 Details of reference to a publicly available material(s), such as Pharmacopeia, for the details about the efficacy of the formula.			0/2 (0)	
	(patent) 11a.1A4 Details of reference to a publicly available material(s), such as Pharmacopeia, for the details about the safety of the formula.			0/2 (0)	
	(patent) 11a.1A5 Details of reference to a publicly available material(s), such as Pharmacopeia, for the details about the quality control of the formula.			1/2 (50)	
	(patent) 11a.1A6 Detail of the proprietary product name (ie, brand name)			2/2 (100)	
	(patent) 11a.1A7 Detail of name of manufacturer.			2/2 (100)	
	(patent) 11a.1A8 Detail of lot number.			1/2 (50)	
	(patent) 11a.1A9 Detail of production data and expiry date.			0/2 (0)	
	(patent) 11a.1A10 Detail of name and content of added materials			1/2 (50)	
	(patent) 11a.1A11 Detail of whether any additional quality control procedures will be conducted.			0/2 (0)	
	(patent) 11a.1A12 Statement of whether the patent proprietary CHM formula utilized in the study is identical to the publicly available reference.			0/2 (0)	
	Number of protocols with acupuncture intervention	4	7	4	8
	11a.1B1. Detail of treatment environment.	3/4 (75)	5/7 (71)	2/4 (50)	1/8 (13)
	11a.1B1A. Detail of participant posture.	2/4 (50)	3/7 (43)	1/4 (25)	5/8 (63)
	11a.1B2. Detail of number of needle insertions per subject per session (mean and range if possible).	3/4 (75)	3/7 (43)	3/4 (75)	8/8 (100)
	11a.1B3. Detail of location of acupoints (uni/bilateral).	3/4 (75)	2/7 (29)	2/4 (50)	6/8 (75)
	11a.1B3A. Name of all acupoints presented in Chinese (Pinyin) and international code.	4/4 (100)	2/7 (29)	2/4 (50)	8/8 (100)
	11a.1B4. Detail of angle of insertion.	2/4 (50)	0/7 (0)	0/4 (0)	1/8 (13)
	11a.1B4A. Detail of depth of insertion, which should be presented in a specified unit of measurement or on a particular tissue level.	3/4 (75)	3/7 (43)	2/4 (50)	4/8 (50)
	11a.1B5. Detail of response sought (eg, de qi or muscle twitch response).	4/4 (100)	4/7 (57)	4/4 (100)	7/8 (88)
	11a.1B6. Detail of needle stimulation (eg, manual, electrical). If electroacupuncture apparatus will be utilized, the brand, manufacturer, and frequency should be indicated.	2/4 (50)	5/7 (71)	4/4 (100)	5/8 (63)
	11a.1B7. Detail of needle retention time.	4/4 (100)	6/7 (86)	4/4 (100)	7/8 (88)
	11a.1B8A. Detail of needle, including diameter, length.	3/4 (75)	6/7 (86)	4/4 (100)	7/8 (88)
	11a.1B8B. Detail of manufacturer.	4/4 (100)	4/7 (57)	2/4 (50)	6/8 (75)
	11a.1B8C. Detail of needle material.	3/4 (75)	3/7 (43)	2/4 (50)	2/8 (25)
	11a.1B9. Detail of number of treatment sessions.	4/4 (100)	7/7 (100)	4/4 (100)	8/8 (100)
	11a.1B10. Detail of frequency and duration of treatment sessions.	4/4 (100)	6/7 (86)	4/4 (100)	8/8 (100)
	Number of protocols that used placebo control	3	6	11	2
	Placebo control 11a.2A1 Name and dosage of each ingredient.	0/3 (0)	1/6 (17)	3/11 (27)	0/2 (0)
	11a.2A2. Description of the similarity of placebo with intervention (eg, color, smell, taste, appearance, and packing).	2/3 (67)	5/6 (83)	8/11 (73)	2/2 (100)
	11a.2A3. Quality control and safety surveillance, if any.	0/3 (0)	1/6 (17)	1/11 (9)	0/2 (0)
	11a.2A4. Administration route, dosage, and regimen.	2/3 (67)	2/6 (33)	6/11 (55)	1/2 (50)
	11a.2A5. Production information: when, where, how, and by whom the placebo was produced.	0/3 (0)	1/6 (17)	0/11 (0)	0/2 (0)
	Number of protocols with CHM active control	0	0	0	0
	CHM active control 11a.2A1 If a CHM formula was used, refer to the recommendations of 11a.1A.				
	Pharmacological active control Number of protocols that used a chemical agent as control	0	7	1	2
	Pharmacological active control 11a.2A2 If a chemical agent was used, the name.		7/7 (100)	1/1 (100)	2/2 (100)
	11a.2A3 If a chemical agent was used, the administration route.		1/7 (14)	0/1 (0)	2/2 (100)
	11a.2A4 If a chemical agent was used the dosage		6/7 (86)	0/1 (100)	2/2 (100)
	11a.2A5 If a chemical agent was used the regime.		7/7 (100)	1/1 (100)	2/2 (100)
	11a.2B1. Acupuncture Blank/waitlist control State any special arrangement(s) in pre-treatment, treatment, and post-treatment periods corresponding to the experimental intervention (eg, examinations in pre-treatment period, unaltered lifestyle and medication in treatment period, and compensatory interventions in post-treatment period).		2/2 (100)	2/3 (67)	3/5 (60)
	11a.2B2. Acupuncture Sham acupuncture or acupuncture-like control State the comparability of the sham acupuncture or acupuncture-like control and comprehensively provide details as for the recommendations of 11a.1B.	2/2 (100)	1/3 (33)	2/3 (67)	2/4 (50)
	11d.2 Descriptions of other interventions that will be administrated to experimental and/or control groups are recommended (eg, rescue interventions), with enough details to allow replication.	2/8 (25)	8/15 (53)	5/18 (28)	1/12 (8)
Outcomes	12a. Provide the rationale of TCM-related indexes as outcomes (eg, the change of degree and scope of symptoms and signs related to Pattern differentiation).	0/8 (0)	3/15 (20)	2/18 (11)	2/12 (17)
	12b1. Provide the details of the TCM-related outcomes assessment, including the measuring methods and standard (eg, frequency, severity rating scale of symptoms and signs, verified Pattern questionnaire, time points for assessment, and corresponding rationale.)	1/8 (13)	4/15 (27)	0/18 (0)	2/12 (17)
	12b2. Provide the details of the TCM-related outcomes assessment including assessor qualification (eg, relevant assessment experience, years in clinical practice).	0/8 (0)	3/15 (20)	0/18 (0)	0/12 (0)
	12b3. Provide the details of the TCM-related outcomes assessment including methods used to enhance the quality of assessment (eg, multiple repeated observation, training of assessors).	0/8 (0)	4/15 (27)	1/18 (6)	0/12 (0)
	18a.1 Protocol was targeted on TCM Pattern, or a WM-defined disease with a specific TCM Pattern baseline data about TCM Pattern was provided.	1/8 (13)	3/15 (20)	0/18 (0)	2/12 (17)
Data collection methods	31a1. Plan for raw data sharing with detail of when the data will become available.	1/8 (13)	1/15 (7)	0/18 (0)	1/12 (8)
	31a2. Plan for raw data sharing with detail of how the data will be shared.	1/8 (13)	1/15 (7)	2/18 (11)	0/12 (0)
Dissemination Policy	31a3. Plan for raw data sharing with detail of what data in particular will be shared.	1/8 (13)	1/15 (7)	2/18 (11)	1/12 (8)
	31a4. Plan for raw data sharing with detail of who could acquire the data.	1/8 (13)	5/15 (33)	2/18 (11)	1/12 (8)
	31a5. Plan for raw data sharing with detail of through what access data will be shared.	0/8 (0)	0/15 (0)	1/18 (6)	1/12 (8)

## Discussion

None of the 53 protocols met full concordance. Overall, protocols in TCQ had the lowest rates of concordance compared to protocols in acupuncture and CHM (Table 3). Protocols in acupuncture had relatively more moderate to high rates of concordance in terms of reporting of intervention administration, safety, and quality control. There was notably very low concordance in the reporting of quality control, safety, and administration of fixed and individualized CHM formulas. There were no standardized criteria for intervention reporting specific to the design of TCQ interventions. There was notably very low concordance in the reporting of TCM diagnostic patterns in terms of patient population, patient recruitment, eligibility and diagnostic criteria, and study objectives; TCM theory in the study and intervention rationale; the rationale for selecting corresponding comparators and their success of blinding; the possible interactions between WM and TCM interventions; qualifications and experience of participant screeners, outcome assessors, and data analysts; and TCM-related outcome assessments.

### Primary Reasons for the Low Concordance With SPIRIT-TCM Extension 2018

We assessed the extent of concordance with the SPIRIT-TCM Extension 2018, in clinical protocols of acupuncture, CHM, and TCQ interventions for cancer care and found significant gaps between what was recommended and what was reported. The completeness and transparency of clinical protocols ensure research reproducibility and assist researchers, academics, editors, and peer reviewers, as well as the general readership, to understand, interpret, and critically appraise the design and risk of bias for the planned clinical trials.^72^ Over the past decade, a system of reporting guidelines for TCM clinical trials has been established, addressing various TCM interventions.^73^ Guidelines have been developed on trial registration, protocol design, results publication, evidence synthesis and clinical practice, yet their application remains suboptimal.^73^ It is necessary to explore the possible reasons for the overall low concordance to improve the quality of trials in acupuncture, CHM, and TCQ in cancer care. The common reasons are as below.

### The Difference Between TCM and WM Theoretical Framework

The difference in theoretical framework between TCM and WM may be a considerable factor contributing to the gap in concordance between what was recommended by SPIRIT-TCM Extension 2018 and what has been reported in published protocols. Despite the recommendations of the SPIRIT-TCM Extension 2018, the differences between TCM and WM in clinical practices, principles and treatment philosophies continue to pose challenges for researchers designing TCM intervention trials to satisfy the essential requirements of both epistemologies. Notably, individualization in TCM pattern diagnosis and TCM-related outcome assessment stands as a cornerstone of TCM practice. Yet it remains a challenge to integrate this clinically important feature into TCM cancer research. This was reflected in our findings which identified the lowest concordance in the reporting of TCM diagnostic patterns in terms of patient population, patient recruitment, eligibility and diagnostic criteria, study objectives, and TCM-related outcome assessments. One possible explanation is that researchers may tend to prioritize meeting the standardization requirements of the relatively more rigid epistemology of WM research over TCM principles of pattern differentiation and individualized intervention. This approach may be considered more likely to gain widespread recognition and acceptance. Another barrier lies in the challenge of communicating TCM theorems to reviewers and readers from non-TCM backgrounds. It is evident that some studies have applied a TCM diagnostic framework when selecting acupoints, CHM and TCM-related parameters.^30,43^ However, when reporting, the researchers attributed these choices to their “previous experience” in consideration of the unfamiliarity with TCM principles within the broader scientific community. Hence, in an effort to avoid potential resistance when adopting novel methodologies that accommodate TCM features or when articulating TCM rationales, researchers may choose to exclude these TCM characteristics from their study design or reporting.

To overcome the dissonance between the 2 theoretical frameworks, we recommend that researchers who are undertaking research in integrative care better understand the features of each discipline. A researcher from a TCM background needs to be fully aware of the requirements of the biomedical research design, while a researcher from a WM background conducting TCM research, needs to understand the philosophy, principles, and practices of TCM research. In this way, TCM researchers may develop novel methodologies capable of integrating the essential requirements of TCM and WM theoretical frameworks and bridging the gap between the 2 epistemologies.

Additionally, to help familiarize the wider scientific community with the critical components of TCM treatment principles, we encourage researchers to report brief explanations of the TCM rationale behind the pattern diagnosis and treatment choices, and provide references to more sophisticated discussions of TCM principles where necessary.

### The Practicality of Incorporating TCM Features in Trials

Another practical consideration when incorporating TCM pattern diagnosis into trial design is the inter-rater reliability of TCM diagnostics, which is found to be suboptimal.^74^ Consequently, researchers may be concerned that the inclusion of TCM diagnosis in the study could lead to disagreements among clinical assessors, potentially compromising the reliability and replicability of study outcomes. One potential solution is to establish strict qualification requirements for study assessors and provide group training before the trial, as this has been shown to improve inter-rater agreement.^74^

### Researcher’s Consideration of Importance and Relevance

Researchers of TCM may come to appreciate the importance of not rigidly adhering to every checklist item in SPIRIT-TCM Extension 2018. Due to the dissonance caused by the differences between TCM and WM theoretical frameworks, researchers may choose not to follow certain recommendations from the SPIRIT-TCM Extension 2018, leading to a misconception that these items are not important in the design of TCM clinical research. Researchers of TCM may not be used to designing TCM research under the framework of Western research methods, and this may have contributed to the low concordance in reporting the rationale for selecting corresponding comparators and their success of blinding; the possible interactions between WM and TCM interventions; qualifications and experience of participant screeners, TCM intervention providers and outcome assessors, and data analysts. Moreover, in China, cultural factors may influence judgments about risk, safety, and quality control in CHM research.^75^ While risk assessment is indeed widely recognized as a critical consideration in clinical research, in China, where CHM has an extensive history in clinical practice, there is a cultural assumption regarding the safe usage of CHM based on cultural familiarity and historical use, in contrast to pharmaceutical drugs.^75^ This influence of conception may contribute to our findings of low concordance in the reporting of quality control, safety, and administration of CHM formulas. To improve the understanding of what TCM researchers may consider important and relevant in clinical trial protocols, we will be undertaking a Delphi survey in the future to explore their considerations in greater depth.

### The Incompleteness of the SPIRIT-TCM Extension 2018

The SPIRIT-TCM Extension 2018 may not be complete enough as a set of criteria to encompass the particularities of TCM clinical research in cancer care. Originally based on the SPIRIT 2013, a set of standard recommendations for biomedical rationale-based clinical trials, the SPIRIT-TCM Extension 2018 may prescribe a set of criteria that is too rigid to appropriately integrate the particularities of TCM theory and research design. Our study found very low concordance in the SPIRIT-TCM Extension 2018 checklist item that recommended reporting the rationale for the success of blinding. However, it is important to mention that for TCM interventions such as acupuncture, some researchers have expressed a preference for waitlist control designs over blinded designs, arguing that the former better reflects real-world practice and helps with recruitment.^23^ Moreover, it is worth considering whether blinding is possible in TCQ studies. Importantly, the SPIRIT-TCM Extension 2018 does not have a standardized set of criteria for intervention design specific to TCQ interventions, which have increasing popularity for improving symptom management during cancer treatment and survivorship.^7^ This may have contributed to our finding that TCQ had the lowest rates of concordance compared to protocols in acupuncture and CHM. To develop a more fit-for-purpose set of criteria for designing clinical protocols in acupuncture, CHM, and TCQ in cancer care, we recommend updating the SPIRIT-TCM Extension 2018 with criteria for TCQ intervention, in addition to exploring TCM researcher considerations for other essential criteria.

### Awareness of SPIRIT-TCM Extension 2018

It is unknown whether researchers in TCM clinical interventions for cancer care are aware of the release of the SPIRIT-TCM Extension 2018. Three years have passed since the publication of the SPIRIT-TCM Extension 2018, which was accepted and endorsed through consensus by the TCM academic and research community. A recent study on reporting compliance with SPIRIT 2013 found a positive association between publicly reported journal policy of compliance with the SPIRIT statement and higher reporting quality.^76^ While the current peer-reviewed academic journals have not implemented adherence to the SPIRIT-TCM Extension 2018, a possible avenue for increasing researcher awareness may be through encouraging its endorsement by journals and editors, through the introduction of presubmission checklists, structured online manuscript submission systems and automated manuscript reporting quality checks. Incorporating the SPIRIT-TCM Extension 2018 into the mandatory fields required by clinical trial registries may also be a possible way of promoting awareness of the recommendations.

### Limitations

In this study, we performed a literature search for clinical trial protocols in acupuncture, CHM, and TCQ interventions in cancer care that were published following the publication of the SPIRIT-TCM Extension 2018. Although we assessed a total of 53 trial protocols systematically, the current study still has several limitations. First, our study was limited by the inclusion of only clinical trial protocols published in Chinese and English language. However, the influence is not significant because the majority of clinical trial protocols in acupuncture, CHM, and TCQ are published in these 2 languages. Second, our review only identified a limited sample of 53 protocols. We consider that as more time passes and further protocols are published, future studies may review a greater sample. Thirdly, we consider that the low rate of concordance may be due to the limited time that has passed since the relatively recent release of the SPIRIT-TCM Extension 2018. From reviewing the rate of concordance across each year from 2019 to 2022, there has not been a significant improvement in the reporting quality of clinical trial protocols in acupuncture, CHM, or TCQ in cancer care. Future studies may continue to map concordance over additional years to gain further insight into the impact of the time delay and determine whether the low concordance is indeed related to a lack of researcher awareness. Lastly, this review found no protocols published in Chinese biomedical journals. While we screened 50 articles from Chinese biomedical journals that contained protocol details matching some of our eligibility criteria (measured cancer outcomes and employed TCM interventions), the articles were ultimately excluded as they reported study results and therefore were not clinical research protocols. The findings are consistent with a previous review^77^ in demonstrating that there are no Chinese biomedical journals publishing research protocols. The articles that we found published in Chinese biomedical journals tend to directly proceed to report clinical trial findings without a published protocol. It may be worth recommending to Chinese-language Journal editors and regulators the benefits of protocol publication for improving the quality of journals, particularly emphasizing the inclusion of the publication of TCM cancer care trial protocols as an integral part of medical research.

## Conclusion

The increase in cancer incidence and mortality challenges the global delivery of cancer care. The application of TCM in cancer care is an area of enormous interest and the development of a comprehensive evidence base is a priority. To guide the design and reporting of TCM clinical trial protocols, the SPIRIT-TCM Extension 2018 was created and published in 2019. We found that the reporting concordance with SPIRIT-TCM Extension 2018 is low, and there remains a poor integration of TCM characteristics with standardized research design which may impact the ultimate validity and possibly the safety of combining both treatment modalities and philosophical approaches to treatment. We intend to undertake a future Delphi survey to identify opportunities for further expansion or elaboration on the specific guidance for TCM cancer care clinical trial protocols. We recommend that researchers who are undertaking research in integrative care understand the features of each discipline, and in this way develop novel methodologies capable of integrating, at an epistemological level, the essential requirements of TCM and WM theoretical philosophies.
